# Characteristics of patients requiring tracheostomy following extracorporeal cardiopulmonary resuscitation for out-of-hospital cardiac arrest^☆^

**DOI:** 10.1016/j.resplu.2025.100911

**Published:** 2025-02-20

**Authors:** Shutaro Isokawa, Toru Hifumi, Eiki Iida, Sohma Miyamoto, Kasumi Shirasaki, Tasuku Hada, Akihiko Inoue, Tetsuya Sakamoto, Yasuhiro Kuroda, Norio Otani, The SAVE-J II study group

**Affiliations:** a Department of Emergency and Critical Care Medicine, St. Luke’s International Hospital, Tokyo, Japan; b Department of Emergency and Critical Care Medicine, Hyogo Emergency Medical Center, Kobe, Japan; c Department of Emergency Medicine, Teikyo University School of Medicine, Tokyo, Japan; d Department of Emergency Medicine, Kagawa University School of Medicine, Kagawa, Japan

**Keywords:** Extracorporeal cardiopulmonary resuscitation, Out-of-hospital cardiac arrest, Tracheostomy

## Abstract

**Aim:**

This study aimed to describe the characteristics of patients requiring tracheostomy following extracorporeal cardiopulmonary resuscitation (ECPR) for out-of-hospital cardiac arrest (OHCA) using real-world data from a multicenter registry.

**Methods:**

This was a secondary analysis of the SAVE-J II study, a retrospective multicenter registry study in Japan. Patients with OHCA aged ≥18 years who underwent ECPR between January 2013 and December 2018 were included. Participants were classified into the tracheostomy and non-tracheostomy groups, with the tracheostomy group further categorized into early (≤10 days) and late (>10 days) subgroups. Survival and favorable neurological outcome at hospital discharge were the primary outcomes.

**Results:**

Overall, this study included 1,910 patients with a median age of 61 (interquartile range [IQR], 49–69) years, of whom 1,610 (82.6%) were male. Of the participants, 276 (14.5%) underwent tracheostomy, with 224 (81.2%) and 44 (15.9%) surviving to discharge and achieving favorable neurological outcomes at hospital discharge, respectively. The median duration to tracheostomy was 10 (IQR, 8–14) days, with 98% of tracheostomies performed following extracorporeal membrane oxygenation (ECMO) weaning. The early tracheostomy group accounted for 145 patients (54.7%). The early and late tracheostomy subgroups showed no significant differences in survival or favorable neurological outcomes at discharge.

**Conclusions:**

Following ECPR, 14.5% of the patients underwent tracheostomy, with the majority performed following ECMO weaning. Although the survival rate at discharge among these patients was 81.2%, only 15.9% exhibited favorable neurological outcomes. To explore the long-term outcomes of patients treated with ECPR for OHCA, future studies are needed.

## Introduction

The application of extracorporeal cardiopulmonary resuscitation (ECPR) is increasing in patients with out-of-hospital cardiac arrest (OHCA) who are refractory to conventional advanced cardiac life support.^1^, ^2^ Previous studies have reported that ECPR is associated with improved survival and neurological outcomes in patients presenting with OHCA.^3^, ^4^, ^5^ However, the expanded application of ECPR may result in a higher demand for post-resuscitation management and a corresponding increase in the number of patients requiring tracheostomy within intensive care. Few studies have comprehensively investigated the clinical characteristics of patients requiring tracheostomy following ECPR, and to select patients for ECPR and optimize post-resuscitation management, understanding the characteristics of these patients may be essential. Therefore, this study aimed to investigate the characteristics of patients requiring tracheostomy following ECPR for OHCA using a dataset from 36 institutions in Japan.

## Methods

### SAVE-J II study

The SAVE-J II study was a multicenter retrospective registry study conducted in Japan, with 36 participating institutions. This study aimed to investigate the outcomes of patients with OHCA who received ECPR.^6^ It was registered at the University Hospital Medical Information Network Clinical Trials Registry and the Japanese Clinical Trial Registry (registration number: UMIN000036490). The Institutional Review Board of Kagawa University (approval number: 2018-110) and each participating institution, including St. Luke’s International Hospital (approval number: 18-R188), approved this study.

### Study design and participants

This study performed a secondary analysis of the SAVE-J II study,^6^ which encompassed patients aged ≥18 years who presented with OHCA, were admitted to the emergency department between January 1, 2013 and December 31, 2018, and received ECPR (resuscitation for cardiac arrest with venoarterial extracorporeal membrane oxygenation [VA-ECMO]). The following were the exclusion criteria: patients who received VA-ECMO following intensive care unit (ICU) admission, those who were withdrawn after cannulation owing to the return of spontaneous circulation (ROSC), those who achieved ROSC at hospital arrival and ECMO initiation, those with unknown outcomes and cannula insertion failure, and those without records of tracheostomy. This secondary analysis was conducted with the approval of the Institutional Review Board of St. Luke’s International Hospital (approval number: 23-R079), and the requirement for informed consent was waived. All procedures were performed in accordance with the ethical standards of the review board of St. Luke’s International Hospital on human experimentation and with the Helsinki Declaration of 1975.

### Data collection

The following data of patients who received ECPR in the SAVE-J II study were collected: age, sex, comorbidities, baseline performance status (PS),^7^ occurrence of witnessed cardiac arrest and bystander cardiopulmonary resuscitation (CPR), initial cardiac rhythm at the scene and before ECMO initiation, time course, percutaneous catheter intervention (PCI), etiology of cardiac arrest, length of ICU stay, length of hospital stay, length of ventilator management days, do-not-attempt-resuscitation (DNAR) order during the stay in the participating hospital, neurological outcomes at hospital discharge, and days from admission to tracheostomy and ECMO weaning.

### Definitions

Time from emergency call to ambulance arrival was defined as the time from calling emergency medical services to hospital arrival. Estimated low-flow time was defined as the time from cardiac arrest to the establishment of ECMO when the cardiac arrest location was in an ambulance and the time from calling an ambulance to the establishment of ECMO when cardiac arrest occurred in another location.^6^ Early tracheostomy was defined as placement within the first 10 days of ventilation, whereas late tracheostomy was defined as placement on day 11 or later following ventilation initiation.^8^

### Outcome measures

In this study, hospital mortality and neurological outcome at hospital discharge were the primary outcomes, and discharge disposition and the timing of tracheostomy were the secondary outcomes.

### Statistical analysis

Continuous variables were presented as medians with interquartile ranges (IQRs), whereas categorical variables were expressed as numbers and percentages (%). The Mann–Whitney–Wilcoxon test and chi-squared test were used for comparing continuous and categorical variables between the two groups, respectively. *P*-values of <0.05 were considered statistically significant. All statistical analyses were performed using JMP Pro 18.0.1 (SAS Institute, Cary, NC, USA). Missing data were not replaced or estimated.

## Results

**Fig. 1:**
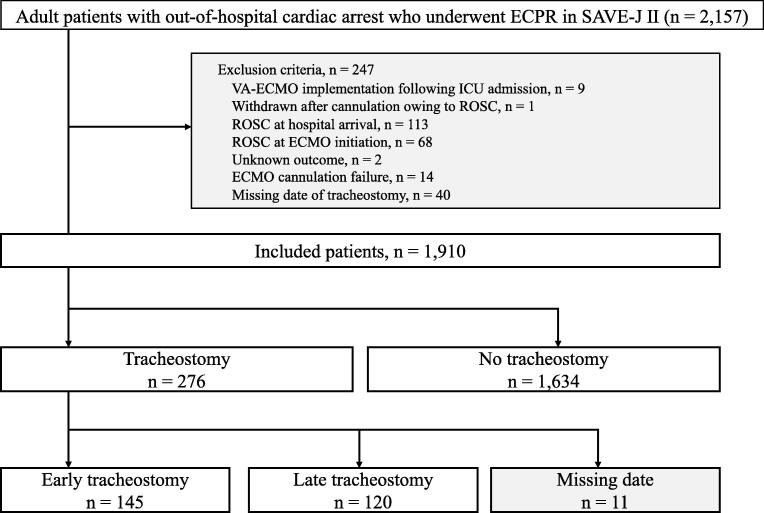
**Patient selection flow chart.** ECPR, extracorporeal cardiopulmonary resuscitation; VA-ECMO, venoarterial extracorporeal membrane oxygenation; ICU, intensive care unit; ROSC, return of spontaneous circulation.

The patient selection flow chart is shown in Fig. 1. Of the 2,157 adult patients with OHCA who received ECPR in the SAVE-J II study, 1,910 were included in this study, of whom 276 (14.5%) underwent tracheostomy during hospitalization (tracheostomy group) and 1,634 (85.5%) did not undergo tracheostomy (no tracheostomy group). Moreover, the tracheostomy group was categorized into the early and late tracheostomy subgroups, comprising 145 (52.5%) and 120 (43.5%) patients, respectively.

### Patients’ baseline characteristics

The study population comprised 1,910 patients, with a median age of 61 (IQR, 49–69) years, and 82.6% were male. The following were the prevalence rates of comorbidities among the patients: hypertension (29.9%), diabetes mellitus (19.1%), dyslipidemia (10.7%), cardiovascular disease (23.2%), stroke (6.6%), and chronic kidney disease (5.0%). The following were the distributions of PS before admission: PS0, 1,644 patients (89.2%); PS1, 161 patients (8.7%); PS2, 28 patients (1.5%); PS3, 10 patients (0.5%); and PS4, 1 patient (0.1%). Among these patients, 1,467 (77.0%) involved witnessed cardiac arrest, 1,073 (57.0%) received bystander CPR, and 1,211 (64.0%) presented with a shockable rhythm on the initial cardiac rhythm. The median time from emergency call to ambulance arrival was 32 (IQR, 26–40) min, and the median estimated low-flow time was 55 (IQR, 45–68) min. PCI was performed in 766 patients (41.1%). Reintubation was 15 patients (1.5%). The median ICU stay was 3 (IQR, 1–10) days, the median mechanical ventilation duration was 3 (IQR, 1–8) days, and the median hospital stay was 3 (IQR, 1–18) days. DNAR orders were issued for 558 patients (30.7%). At discharge, 265 patients (13.9%) had a favorable neurological outcome, and 504 patients (26.4%) were survival discharge (Table 1).

**Table 1:** Characteristics and comparisons between patients with and without tracheostomya.

	All N = 1,910	Tracheostomy n = 276	Non-tracheostomy n = 1,634	p-value
Age, years	61 (49–69)	61 (50–67)	61 (49–69)	0.393
Sex, male	1,610 (82.6)	233 (84.4)	1,343 (85.2)	0.367
Comorbidity				
Hypertension	571 (29.9)	81 (29.4)	490 (30.0)	0.829
Diabetes mellitus	364 (19.1)	46 (16.7)	318 (19.5)	0.274
Dyslipidemia	204(10.7)	38 (13.8)	166 (10.2)	0.073
Cardiovascular disease	444 (23.2)	58 (21.0)	386 (23.6)	0.343
Cerebrovascular disease	126 (6.6)	11 (4.0)	84 (5.1)	0.414
Chronic renal failure	95 (5.0)	7 (3.1)	15 (5.6)	0.223
Performance status				0.079
PS0	1,644 (89.2)	238 (87.8)	1,406 (89.4)	
PS1	161 (8.7)	27 (10.0)	134 (8.5)	
PS2	28 (1.5)	5 (1.9)	23 (1.5)	
PS3	10 (0.5)	0(0)	10 (0.6)	
PS4	1 (0.1)	1 (0.4)	0(0)	
Witnessed cardiac arrest	1,467 (77.0)	219 (79.6)	1,248 (76.7)	0.277
Bystander CPR	1,073 (57.0)	140 (51.5)	933 (58.0)	0.045
Initial cardiac rhythm				<0.001
Shockable	1,211 (64.0)	206 (75.7)	1,005 (62.1)	
PEA	497 (26.3)	45 (16.5)	452 (27.9)	
Asystole	183 (9.7)	21 (7.7)	162 (10.0)	
Time from emergency call to ambulance arrival (min) b	32 (26–40)	29 (24–36)	33 (27–40)	<0.001
Estimated low-flow time (min) c	55 (45–68)	47 (39–59)	56 (47–69)	<0.001
Rhythm before ECMO initiation				<0.001
Shockable	964 (51.0)	178 (64.5)	786 (48.7)	
PEA	627 (33.2)	78 (28.2)	549 (34.4)	
Asystole	298 (15.8)	20 (7.2)	278 (17.2)	
Percutaneous catheter intervention	766 (41.1)	134 (49.3)	632 (39.7)	0.003
Length of ICU stay, days	3 (1–10)	15 (11–22)	2 (1–6)	<0.001
Length of mechanical ventilation, days	3 (1–8)	16 (11–25)	2 (1–5)	<0.001
Length of hospital stay, days	3 (1–18)	40 (24–63)	2 (1–8)	<0.001
Reintubation	28 (1.5)	13 (5.0)	15 (0.9)	<0.001
DNAR order	558 (30.7)	56 (21.2)	502 (32.4)	<0.001
Cerebral Performance Category (CPC)				<0.001
CPC1	190 (9.9)	25 (9.0)	165 (10.0)	
CPC2	75 (3.9)	19 (6.9)	56 (3.4)	
CPC3	79 (4.1)	46 (16.7)	33 (2.0)	
CPC4	160 (8.4)	134 (48.6)	26 (1.6)	
CPC5	1,406 (73.6)	52 (18.8)	1,354 (82.9)	
Favorable neurological outcomes at hospital discharge	265 (13.9)	44 (15.9)	221 (13.5)	0.283
Survival at hospital discharge	504 (26.4)	224 (81.2)	280 (17.1)	<0.001
Discharge disposition				<0.001
Home	127 (6.8)	11 (4.1)	116 (7.2)	
Rehabilitation hospital	238 (12.7)	155 (57.6)	83 (5.2)	
Acute care hospital	62 (3.3)	24 (8.9)	38 (2.4)	
Hospice	3 (0.2)	0 (0)	3 (0.2)	
Nursing home	4 (0.2)	4 (1.5)	0 (0)	
Other	38 (2.0)	23 (8.5)	15 (0.9)	
Death	1,406 (74.9)	52 (19.3)	1,354 (84.1)	

CPR, cardiopulmonary resuscitation; ECMO, extracorporeal membrane oxygenation; ICU, intensive care unit; DNAR, do-not-attempt-resuscitation; CPC, Cerebral Performance Category.

Missing date: age, 1; performance status, 66; witnessed cardiac arrest, 7; bystander CPR, 29; initial cardiac rhythm, 19; time emergency call to ambulance arrival, 31; estimated low-flow time, 103; rhythm before ECMO, 21; percutaneous coronary intervention, 44; cardiac arrest etiology, 2; length of ICU stay, 14; length of ventilator management, 28; length of hospital stay, 9; Reintubation, 16; discharge disposition, 32.

a Data are presented as medians (interquartile ranges) for continuous variables and n (%) for categorical variables.

b Time from emergency call to ambulance arrival is the time from calling emergency medical services to hospital arrival.

c Estimated low-flow time is the time from cardiac arrest to the establishment of ECMO when the location was in an ambulance and the time from calling an ambulance to the establishment of ECMO when cardiac arrest occurred in another location.

### Comparisons of the characteristics between the tracheostomy and non-tracheostomy groups

Of the patients, 276 (14.5%) and 1,634 (85.5%) were included in the tracheostomy and non-tracheostomy groups, respectively. Univariate analysis comparing the tracheostomy and non-tracheostomy groups revealed significant differences in terms of bystander CPR, initial cardiac rhythm, time from emergency call to ambulance arrival, estimated low-flow time, rhythm before ECMO initiation, PCI, length of ICU stay, mechanical ventilation duration, hospital stay, Reintubation, DNAR orders, and discharge disposition. No significant differences were observed regarding age, sex, comorbidities, PS, witnessed cardiac arrest, or favorable neurological outcome at hospital discharge (44 patients [15.9%] vs. 221 patients [13.5%], *p* < 0.283). The tracheostomy group had a significantly higher survival at discharge than the non-tracheostomy group (224 patients [81.2%] vs. 280 patients [17.1%], *p* < 0.001). Furthermore, patients in the tracheostomy group were more frequently discharged to rehabilitation hospitals than those in the non-tracheostomy group (155 patients [57.6%] vs. 83 patients [5.2%], *p* < 0.001) (Table 1).

### Timing of tracheostomy

**Fig. 2:**
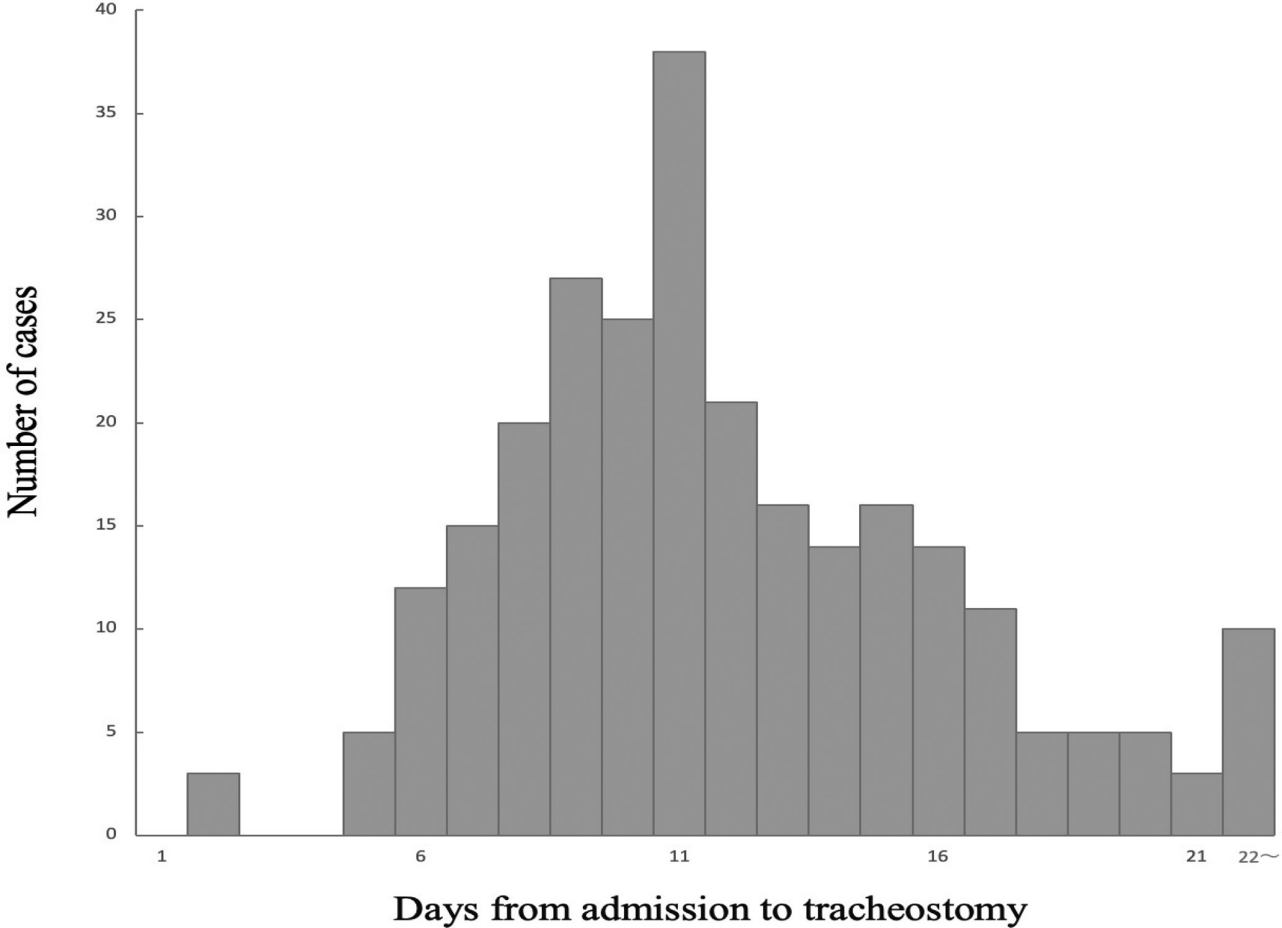
Duration from admission to tracheostomy.

**Fig. 3:**
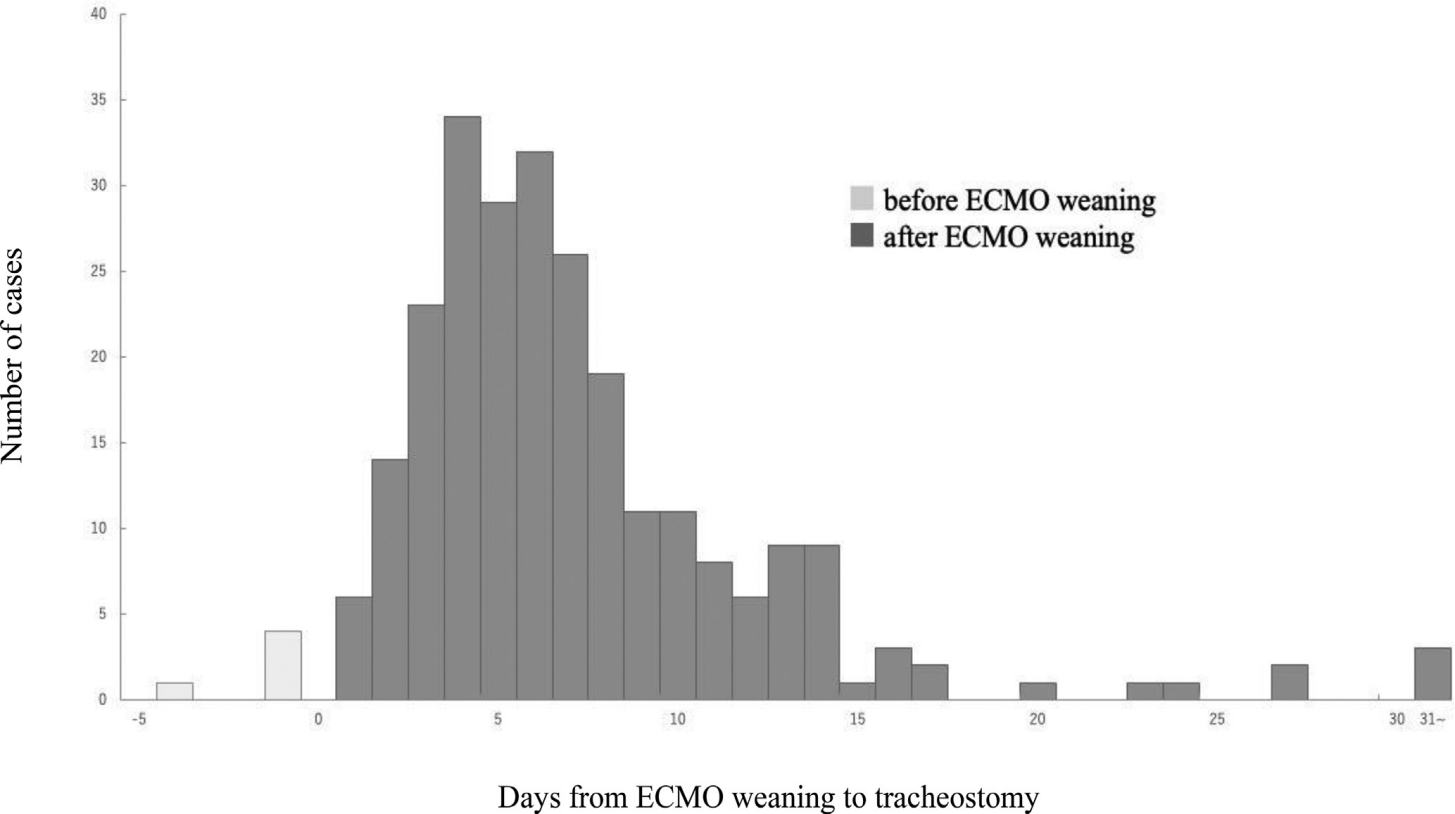
**Days from ECMO weaning to tracheostomy.** ECMO, extracorporeal membrane oxygenation.

The distribution of days from admission to tracheostomy is shown in Fig. 2. The median time was 10 (IQR, 8–14) days, with a range of 1–91 days. Furthermore, as shown in Fig. 3, tracheostomy is performed during ECMO in only 5 patients. In the remaining 251 patients (98%), tracheostomy was performed following ECMO weaning, with a median interval of 6 (IQR, 4–9) days.

### Comparisons of the characteristics between the early and late tracheostomy subgroups

Of the patients who underwent tracheostomy, 145 (54.7%) were categorized into the early tracheostomy group. Comparisons between the early and late tracheostomy subgroups showed that sex distribution, duration of ECMO support, duration from ECMO weaning to tracheostomy, length of ICU stay, mechanical ventilation duration, and length of hospital stay were significantly longer in the late tracheostomy subgroup. However, no significant differences in neurological outcomes and survival at hospital discharge were noted between the early and late tracheostomy subgroups (21 patients [14.5%] vs. 21 patients [17.5%] and 117 patients [80.7%] vs. 98 patients [81.6%]), respectively. Additionally, the rate of reintubation did not differ significantly between the two groups. Regarding the etiology of cardiac arrest, the late tracheostomy subgroup showed a slight tendency toward a higher incidence of acute coronary syndrome (ACS) (73 patients [50%] vs. 73 patients [61%]) and lower incidence of arrhythmia (29 patients [20%] vs. 11 patients [9%]) than the early tracheostomy subgroup; however, these differences were not statistically significant (Table 2).

**Table 2:** Characteristics and comparisons between patients with early and late tracheostomy.

	Early tracheostomy n = 145	Late tracheostomy n = 120	p-value
Age, years	61 (49–67)	61 (51–69)	0.445
Sex, male	129 (89.0)	96 (80.0)	0.042
Duration of ECMO support, days	3 (2–4)	4 (4–7)	<0.001
Duration from ECMO weaning to tracheostomy, days	4 (3–6)	9 (7–13)	<0.001
Length of ICU stay, days	12 (10–17)	20 (15–27)	<0.001
Length of mechanical ventilation, days	12 (9–20)	21 (15–33)	<0.001
Length of hospital stay, days	31 (20–53)	52 (34–78)	<0.001
Reintubation	5 (3.6)	7 (6.0)	0.362
Cerebral Performance Category at hospital discharge			0.168
CPC1	13 (9.0)	12 (10.0)	
CPC2	8 (5.5)	9 (7.5)	
CPC3	18 (12.4)	27 (22.5)	
CPC4	78 (53.8)	50 (41.7)	
CPC5	28 (19.3)	22 (18.3)	
Favorable neurological outcomes at hospital discharge	21 (14.5)	21 (17.5)	0.503
Survival at hospital discharge	117 (80.7)	98 (81.6)	0.840
Cardiac arrest etiology			0.456
Acute coronary syndrome	73 (50.3)	73 (60.8)	
Arrhythmia	29 (20.0)	11 (9.1)	
Myopathy	10 (6.9)	6 (5.0)	
Myocarditis	2 (1.4)	4 (3.3)	
Other cardiac causes	4 (2.8)	6 (5.0)	
Pulmonary embolism	4 (2.8)	5 (4.2)	
Drowning	0 (0)	0 (0)	
Drug intoxication	0 (0)	0 (0)	
Primary cerebral disorders	1 (0.7)	1 (0.8)	
Infection	1 (0.7)	1 (0.8)	
Suffocation	0 (0)	1 (0.8)	
Trauma	0 (0)	0 (0)	
Other non-cardiac causes	2 (1.4)	2 (1.7)	
Unknown	4 (2.8)	2 (1.7)	

ICU, intensive care unit; CPC, Cerebral Performance Category.

Missing date: duration of ECMO support, 9; duration from ECMO weaning to tracheostomy, 9; length of ICU stay, 7; length of ventilator management, 7; length of hospital stay, 2; Reintubation, 8.

## Discussion

To our knowledge, this is the first study investigating the characteristics of patients undergoing tracheostomy following ECPR and the timing of tracheostomy using real-world clinical data from a multicenter retrospective registry. Among eligible patients, 14.5% underwent tracheostomy. Although the tracheostomy group demonstrated high survival to discharge (81.2%), favorable neurological outcomes were limited to 15.9%. Additionally, among those with tracheostomy who survived to discharge, only 4.1% were discharged directly to their homes, whereas 57.6% were transferred to rehabilitation hospitals. In comparison, Agarwal et al. reported that among patients with tracheostomies following cardiac arrest, 11.3% and 24.2% were discharged home and to rehabilitation hospitals, respectively, and the 1-year survival and favorable neurological outcomes were observed in 57.6% and 24.2% of patients, respectively.^9^ These findings suggest that survival among patients requiring tracheostomy following ECPR can decrease following hospital discharge, whereas neurological improvement is possible with continued rehabilitation. To investigate the long-term outcomes of patients who underwent ECPR, further research is needed.

The median time to tracheostomy was 10 days, and the procedure was performed following ECMO weaning in almost all patients (98%). In patients receiving ECMO, the optimal timing of tracheostomy continues to spark clinical deliberation. Earlier facilitation of respiratory weaning, reduced sedation requirements, and improved patient comfort constitute the potential advantages of performing tracheostomy during ECMO.^10^ Additionally, early tracheostomy may enable prompt mobilization and rehabilitation, contributing to better long-term recovery in select patients.^11^ Furthermore, recent advancements in surgical techniques and perioperative care have shown that tracheostomy during ECMO can be safely performed under specific conditions, particularly in centers with high levels of expertise.^12^ However, tracheostomy during ECMO possesses inherent risks,^13^ particularly in the context of patients who underwent ECPR and those with ACS who frequently require anticoagulant and antiplatelet therapies, respectively. These medications significantly increase the risk of bleeding, which may cause life-threatening complications during the procedure. Additionally, the hemodynamic instability frequently observed in patients who underwent ECPR may further complicate perioperative care. Conversely, delaying tracheostomy until after ECMO weaning may enable better stabilization of the patient, including the resolution of coagulopathy, improved neurological assessment, and the ability to better predict the patient’s overall prognosis. This study noted that the majority of tracheostomies were performed following ECMO weaning, suggesting a clinical preference for avoiding the heightened risks associated with anticoagulation and antiplatelet therapies during ECMO. Herein, patient-specific factors, including bleeding risk, neurological recovery, and expected ventilator dependence, may contribute to the decision-making process.

This retrospective observational study had several limitations. First, as this study was based on data from a multicenter retrospective registry in Japan, the decision to perform tracheostomy was independently made by each physician or facility. Second, the registry lacks essential data for evaluating the appropriateness of tracheostomy in patients who have undergone ECPR. Specifically, it does not include information on preoperative neuroprognostication, which is crucial for decision-making, the details of tracheostomy techniques (such as surgical vs. percutaneous approaches), or the incidence of complications associated with the procedure. Third, the observed outcomes of patients who underwent tracheostomy group are likely to be affected by immortal time bias. Fourth, the long-term outcomes of patients who underwent tracheostomy following ECPR, including whether they were weaned from the ventilator or the tracheostomy tube could be removed, were not evaluated. Finally, although this registry dataset was collected between 2013 and 2018 and may be considered relatively old, it has contributed to the development of the SAVE-J III study, which is set to begin.^14^

## Conclusion

In patients with OHCA who received ECPR, 14.5% required a tracheostomy. Among these patients, 81.1% survived to discharge; however, only 15.9% exhibited favorable neurological outcomes at discharge. No significant differences were observed regarding survival and neurological outcomes at hospital discharge between the early and late tracheostomy subgroups.

## Funding source

No funding was received for this study.

## Declaration of AI and AI-assisted technologies in the writing process

During the preparation of this manuscript, the first author used ChatGPT (version 4) to assist with English language editing. After using this tool, all authors reviewed and revised the content as needed and takes full responsibility for the final published work.

## CRediT authorship contribution statement

**Shutaro Isokawa:** Writing – review & editing, Writing – original draft, Visualization, Validation, Project administration, Methodology, Investigation, Formal analysis, Data curation, Conceptualization. **Toru Hifumi:** Writing – review & editing, Validation, Supervision, Conceptualization. **Eiki Iida:** Validation, Supervision. **Sohma Miyamoto:** Validation, Supervision. **Kasumi Shirasaki:** Validation, Supervision, Conceptualization. **Tasuku Hada:** Validation, Supervision. **Akihiko Inoue:** Validation, Supervision. **Tetsuya Sakamoto:** Validation, Supervision. **Yasuhiro Kuroda:** Validation, Supervision. **Norio Otani:** Validation, Supervision.

## Data Availability

The datasets used and analyzed during the current study are available from the authors upon reasonable request.

## Declaration of competing interest

The authors declare that they have no known competing financial interests or personal relationships that could have appeared to influence the work reported in this paper.

## Appendix A SAVE-J II study group investigators

The authors would like to thank all members of the SAVE-J II study group who participated in this study: Hirotaka Sawano, M.D., Ph.D. (Osaka Saiseikai Senri Hospital), Yuko Egawa, M.D., Shunichi Kato, M.D. (Saitama Red Cross Hospital), Naofumi Bunya, M.D., Takehiko Kasai, M.D. (Sapporo Medical University), Shinichi Ijuin, M.D., Shinichi Nakayama, M.D., Ph.D. (Hyogo Emergency Medical Center), Jun Kanda, M.D., Ph.D., Seiya Kanou, M.D. (Teikyo University Hospital), Toru Takiguchi, M.D., Shoji Yokobori, M.D., Ph.D. (Nippon Medical School), Hiroaki Takada, M.D., Kazushige Inoue, M.D. (National Hospital Organization Disaster Medical Center), Ichiro Takeuchi, M.D., Ph.D., Hiroshi Honzawa, M.D. (Yokohama City University Medical Center), Makoto Kobayashi, M.D., Ph.D., Tomohiro Hamagami, M.D. (Toyooka Public Hospital), Wataru Takayama, M.D., Yasuhiro Otomo, M.D., Ph.D. (Tokyo Medical and Dental University Hospital of Medicine), Kunihiko Maekawa, M.D. (Hokkaido University Hospital), Takafumi Shimizu, M.D., Satoshi Nara, M.D. (Teine Keijinkai Hospital), Michitaka Nasu, M.D., Kuniko Takahashi, M.D. (Urasoe General Hospital), Yoshihiro Hagiwara, M.D., M.P.H. (Imperial Foundation Saiseikai, Utsunomiya Hospital), Shigeki Kushimoto, M.D., Ph.D. (Tohoku University Graduate School of Medicine), Reo Fukuda, M.D. (Nippon Medical School Tama Nagayama Hospital), Takayuki Ogura, M.D., Ph.D. (Japan Red Cross Maebashi Hospital), Shin-ichiro Shiraishi, M.D. (Aizu Central Hospital), Ryosuke Zushi, M.D. (Osaka Mishima Emergency Critical Care Center), Norio Otani, M.D. (St. Luke’s International Hospital), Migaku Kikuchi, M.D., Ph.D. (Dokkyo Medical University), Kazuhiro Watanabe, M.D. (Nihon University Hospital), Takuo Nakagami, M.D. (Omihachiman Community Medical Center), Tomohisa Shoko, M.D., Ph.D. (Tokyo Women’s Medical University Medical Center East), Nobuya Kitamura, M.D., Ph.D. (Kimitsu Chuo Hospital), Takayuki Otani, M.D. (Hiroshima City Hiroshima Citizens Hospital), Yoshinori Matsuoka, M.D., Ph.D. (Kobe City Medical Center General Hospital), Makoto Aoki, M.D., Ph.D. (Gunma University Graduate School of Medicine), Masaaki Sakuraya, M.D., M.P.H. (JA Hiroshima General Hospital Hiroshima), Hideki Arimoto, M.D. (Osaka City General Hospital), Koichiro Homma, M.D., Ph.D. (Keio University School of Medicine), Hiromichi Naito, M.D., Ph.D. (Okayama University Hospital), Shunichiro Nakao, M.D., Ph.D. (Osaka University Graduate School of Medicine), Tomoya Okazaki, M.D., Ph.D. (Kagawa University Hospital), Yoshio Tahara, M.D., Ph.D. (National Cerebral and Cardiovascular Center), Hiroshi Okamoto, M.D, M.P.H. (St. Luke’s International Hospital), Jun Kunikata, M.D., Ph.D., Hideto Yokoi, M.D., Ph.D. (Kagawa University Hospital).
